# System-on-Chip Integration of a New Electromechanical Impedance Calculation Method for Aircraft Structure Health Monitoring

**DOI:** 10.3390/s121013617

**Published:** 2012-10-11

**Authors:** Hamza Boukabache, Christophe Escriba, Sabeha Zedek, Daniel Medale, Sebastien Rolet, Jean Yves Fourniols

**Affiliations:** 1 Le Laboratoire d'Analyse et d'Architecture des Systèmes (LAAS), French National Center for Scientific research (CNRS), 7 avenue du colonel Roche, F-31077 Toulouse, France; E-Mails: cescriba@laas.fr (C.E.); Medale@laas.fr (D.M.); 2 UPS, INSA, INP, ISAE, Université de Toulouse, F-31077 Toulouse, France; E-Mails: sfzedek@laas.fr (S.Z.); fourniols@laas.fr (J.Y.F.); 3 EADS-Innovation Works, F-31000 Toulouse, France; E-Mail: Sebastien.Rolet@eads.net

**Keywords:** structural health monitoring (SHM), composite aircraft structures, electro-mechanical impedance (EMI), PZT sensors, delaminations, System-on-Chip (SoC)

## Abstract

The work reported on this paper describes a new methodology implementation for active structural health monitoring of recent aircraft parts made from carbon-fiber-reinforced polymer. This diagnosis is based on a new embedded method that is capable of measuring the local high frequency impedance spectrum of the structure through the calculation of the electro-mechanical impedance of a piezoelectric patch pasted non-permanently onto its surface. This paper involves both the laboratory based E/M impedance method development, its implementation into a CPU with limited resources as well as a comparison with experimental testing data needed to demonstrate the feasibility of flaw detection on composite materials and answer the question of the method reliability. The different development steps are presented and the integration issues are discussed. Furthermore, we present the unique advantages that the reconfigurable electronics through System-on-Chip (SoC) technology brings to the system scaling and flexibility. At the end of this article, we demonstrate the capability of a basic network of sensors mounted onto a real composite aircraft part specimen to capture its local impedance spectrum signature and to diagnosis different delamination sizes using a comparison with a baseline.

## 1. Introduction

The constant integration of composite materials into aircraft structures [1] makes maintenance and flaw detection more crucial than ever before for safety reasons and failure prognostics. Unlike metallic alloys, composite structures show after energetic impacts delamination defects without any external visible signs [2], which makes the traditional visual inspection method totally inappropriate. Moreover, the fact those carbon composite materials are dielectric makes widespread inspection techniques based on eddy current useless. The complex intrinsic nature of these materials makes conventional ultrasound nondestructive inspection techniques time consuming and thus very expensive to set on large scale areas [3].

Therefore, in order to monitor these kinds of structures, we have implemented a promising technique called electromechanical impedance measurement into a real-time reconfigurable electronics platform [4]. The aim is to detect and localize delaminations and disbonds in large areas using piezoelectric patches pasted onto the structure's surface. The proposed technique is non-intrusive and is easy to setup. By analyzing the electromechanical impedance of the PZTs we are able to measure the high-frequency local impedance spectrum of the structure, which is highly sensitive to incipient damage [5]. By comparing this measure to a baseline value we are capable to detect any delamination and disbond flaws.

## 2. Methodology

Most of the techniques used in structural health monitoring are based on guided elastic waves. When generated in a host structure, some guided elastic waves such as Lamb waves present the unique advantage of being able to travel long distances with a minimal energy loss [6]. Lamb waves are also called plate waves because they have the ability to probe all the medium thickness surfaces where they propagate. This is only possible when their wavelengths are comparable to the host structure thickness. By knowing the time of flight (TOF) of theses waves and by a temporal identification of the unexpected echoes that are generated by flaws, we are capable to calculate and localize its position. Thus, the combination of three nodes is enough to locate the defect [2].

However, because of the high propagation speed of these waves and their high frequency, these methods need a lot of data processing which makes them time consuming on a CPU with limited resources. In addition, experience shows that some areas such as ribs and stiffeners (*cf.* Figure 7(a)) are very difficult to probe using a rational number of sensors [7].

Vibration sensors and strain sensors are widely used in aviation industries to monitor vibration levels and frequency spectra at critical areas. Eurocopter, one of the main helicopter manufacturers has been using accelerometers for a long time to monitor the main rotor rotation and the blades' integrity [8]. The presence of incipient damage or rotation anomalies may be inferred from changes in the vibration signature. Based on the same technique, a data logger system permanently attached to an Airbus A400M rotating blade was developed by Ratier-Figeac, an aircraft components manufacturer associated to the French CNRS [9,10], to perform a real time modal analysis in order to monitor eventual damages in the blades. However, this method is only efficient in the case where dominant harmonics are always present during normal operation, as the damage diagnosis is based on the appearance of new harmonics or shifts of the existing ones, which indicates that changes occurred in the structure. In addition, the main disadvantage of vibration analysis is its incapability to localize precisely an eventual damage.

In the other hand, electromechanical (E/M) impedance-based structural health monitoring has shown quick success for detecting incipient damage using relatively cheap sensors. The concept was primarily used for non-destructive inspection [11–13] and these studies highlighted the differences between the electromechanical impedance technique and the modal analysis technique. Since that time many studies applied to airplanes [14,15], military helicopters [16] and other structures showed the accuracy of this technique. Generally used at high frequencies (hundreds of kHz and less of MHz) [17,18] to extract a relevant signature, the electromechanical impedance technique is therefore sensitive enough to detect minor damages.

### 2.1. Principle of the Electromechanical Impedance Inspection Technique

According to the piezoelectricity equations, the 1D piezoelectric patch admittance model (Equation (1)) is directly linked to the stiffness of the host structure where the sensor is pasted [19,20]. Therefore, the electromechanical impedance of the PZT patch could be considered as the indicator of the structure integrity:

(1) $$\frac{1}{Z_{\text{PZT}}(f)}=\text{if}.C_{0}\left[1-k_{31}^{2}(\frac{K_{\text{structure}}(f)}{K_{\text{structure}}(f)+K_{\text{PZT}}(f)})\right]$$

where k_31_ is the electro-mechanical coupling factor of the PZT, C_0_ is the capacitance of the sensor; K_structure_ and K_PZT_ are respectively the stiffness of the host structure and the stiffness of the sensor itself. Because the high frequency spectrum is much more sensitive to small damages than lower frequencies, small PZT patches with a high resonance first mode are more suited to E/M impedance techniques.

### 2.2. Conventional Realization of the Method

The electromechanical (E/M) impedance method is an emerging and powerful technique that is based on the measurement of the complex impedance Z_PZT_ (jω) of a piezoelectric patch pasted onto a host structure to detect mechanical flaws.

**Figure 1.**
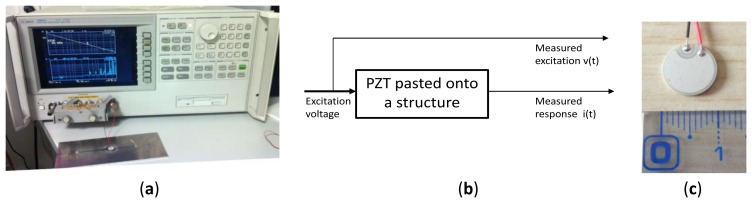
(**a**) Experimental measurement test bench based on an impedance analyzer Agilent 4294A. (**b**) Measurement method principle. (**c**) Photo of the piezoelectric patch, PZT 5H material, 10 mm diameter, 1 mm thickness.

**Figure 2.**
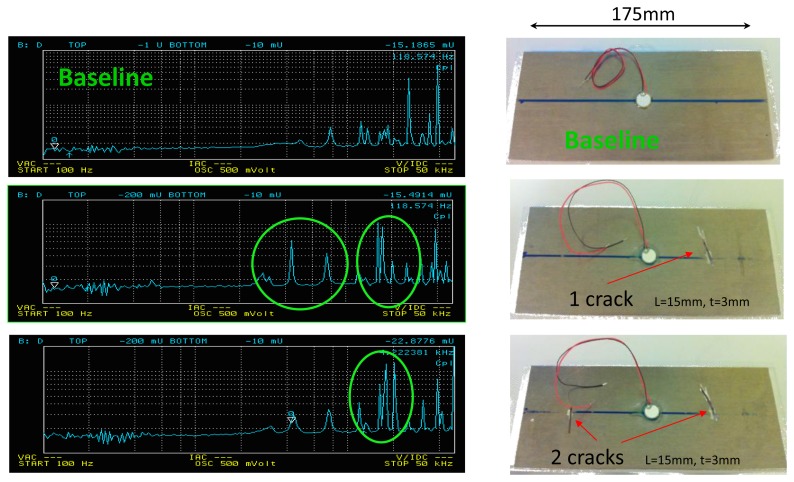
Experimental measurement results recorded using the Agilent 4294A for the same specimen (baseline, and after the introduction of one crack and two cracks). The specimen used for the test is made from an Al2024 certified aeronautics material.

The feasibility and the strength of this method was proven in the literature for concrete structures [21,22] and for different alloys [23] using a piece of laboratory impedance measurement equipment called an impedances analyzer (*cf.* Figure 1). The results presented in Figure 2 were saved using an Agilent 4294A impedance analyzer. The sensors are made from PZT-5H material and are pasted onto a plate of 175 cm × 80 cm × 3 mm dimensions, made from Al2024 aeronautics aluminum certified material. The results show the different responses recorded for the same structure when it was healthy and when the different cracks were introduced. We can easily observe the spike amplitude differences between the plate with one crack and with two cracks.

The impedance analyzer uses pure tone excitation sine waves of different frequencies with fixed numbers of cycles and then measures the sensor response signal. Using a complex sine correlation algorithm implemented in an analog circuit, the impedance analyzer identifies the real and imaginary parts of the probed sensor [24]. This method is time consuming, needs an expensive investment and is unsuitable for an embedded application such as implementation on an airplane.

## 3. Implementation of the EMI Inspection Technique Using a Novel Method

### 3.1. Test Bench

**Figure 3.**
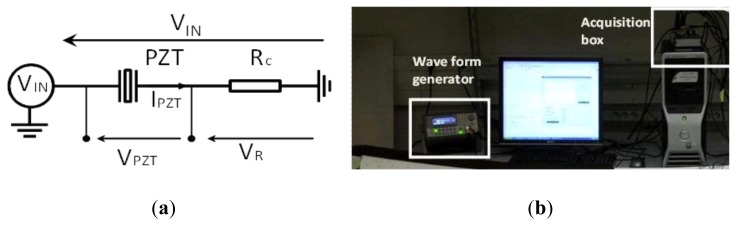
(**a**) Experimental measurement circuit. R_c_ = 10 Ω. (**b**) Test bench setup: An Agilent 33220A arbitrary waveform generator monitored by a central computer over an USB VISA protocol, a PCI National instrument acquisition card of 2.5 Msps.

To make this promising technique implementable, we propose a novel method based on the measurement of the current and voltage sensor response in the time domain to a wideband linear chirp excitation signal and then apply a fast Fourier transform to obtain the complex impedance. The calculation of this impedance is based on the schematic of Figure 3(a).

For the excitation signal, we use a waveform generator (Figure 3(b)) a special linear chirp developed by Giurgiutiu and al [25] that can be synthesized using this general equation:

(2) $$\begin{array}{cc} V_{\text{IN}}(t)=A.e^{j2\pi \left(f_{0}+\frac{\Delta f}{2T}t-\frac{\Delta f}{2}\right)t} & \text{with} \end{array}0\leq t<T$$

A = 10 V is the amplitude of the signal; it sweeps the bandwidth Δf equal to 1.2 MHz centered at f_0_ equal to 600 kHz during the time delay T of 5 s.

**Figure 4.**
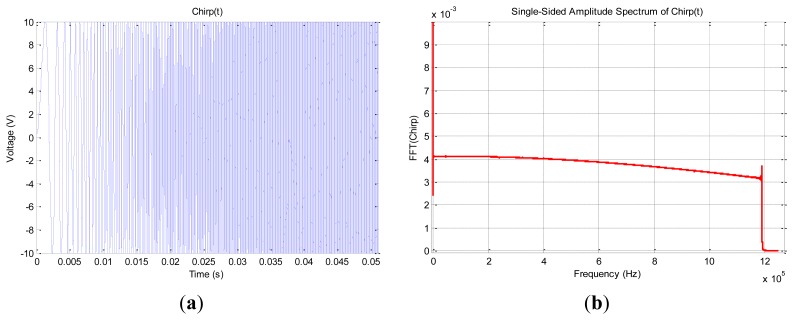
(**a**) Chirp: zoom on temporal signal (**b**) Chirp: Spectral response [0–1.2 MHz].

Using the simple circuit presented in Figure 3(a) we apply the excitation signal to the PZT patch presented in Figure 4 and measure its current response through the resistance Rc. This should be small (∼Ω) to not influence the resonance quality factor of the sensor.

The measurements with the acquisition card (*cf.* Figure 3(b)) of the excitation voltage applied to the piezoelectric patch V_PZT_ (t) and measurement of the current I_PZT_ (t) through the resistance R_c_ allow after a fast Fourier transform the calculation of the complex electro-mechanical impedance of the sensor (Equation (5)):

(3) $$V_{\text{PZT}}(f)=\left(1-\frac{R_{c}}{Z_{\text{PZT}}(f)+R_{c}}\right)V_{\text{IN}}(f)$$

(4) $$I_{\text{PZT}}(f)=\frac{V_{\text{in}}(f)}{Z_{\text{PZT}}(f)+R_{c}}$$

(5) $$Z_{\text{PZT}}(f)=\frac{V_{\text{PZT}}(f)}{I_{\text{PZT}}(f)}$$

### 3.2. Validation of the Method: Detection of Delamination in Composite Structure Skins

**Figure 5.**
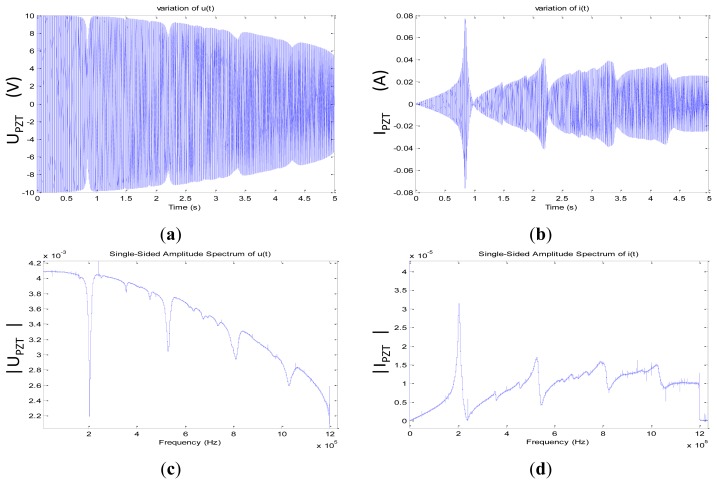
(**a**) V_PZT_ (t): Voltage response of the PZT patch to a chirp of 5 s going from 0 Hz to 1.2 MHz (**b**) I_PZT_ (t): current response of the PZT patch to a chirp of 5 s going from 0 Hz to 1.2 MHz (**c**) V_PZT_ (f) = FFT[V_PZT_ (t)] Voltage spectral response of the PZT to the chirp (**d**) I_PZT_ (f) = FFT[I_PZT_ (t)] current spectral response of the PZT to the chirp.

Using the data presented in Figure 5, we plot the modulus of the complex sensor's impedance by applying Equation (6) and we compare it to the measured |Z_PZT_ (jω)| using a calibrated impedance analyzer Agilent 4294A (*cf.* Figure 1(a)):

(6) $$|Z_{\text{PZT}}(f)|=\frac{\sqrt{\text{Re}{\left[V_{\text{PZT}}(f)\right]}^{2}+Im{\left[V_{\text{PZT}}(f)\right]}^{2}}}{\sqrt{\text{Re}{\left[I_{\text{PZT}}(f)\right]}^{2}+Im{\left[I_{\text{PZT}}(f)\right]}^{2}}}$$

**Figure 6.**
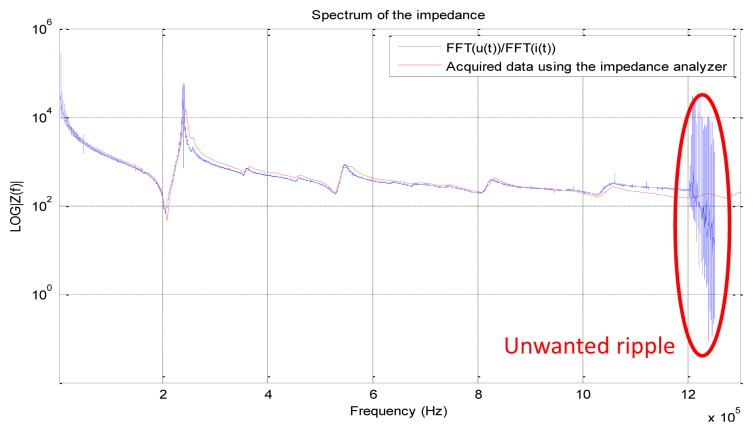
Comparison between the calculated impedance using our experimental setup and the measured one using an Agilent 4294A impedance analyzer. Notice the typical piezoelectric responses at 200 kHz and 240 kHz. It characterizes respectively the resonance and anti-resonance. The unwanted ripples are due to the nature of the spectral response of the applied chirp.

Compared to the data acquired using the Agilent 4294A, the calculated impedance represented in Figure 6 shows many ripples at the edges of the acquisition interval. Theses ripples fortunately appear above 1.2 MHz, which has no consequence for the impedance calculation.

**Figure 7.**
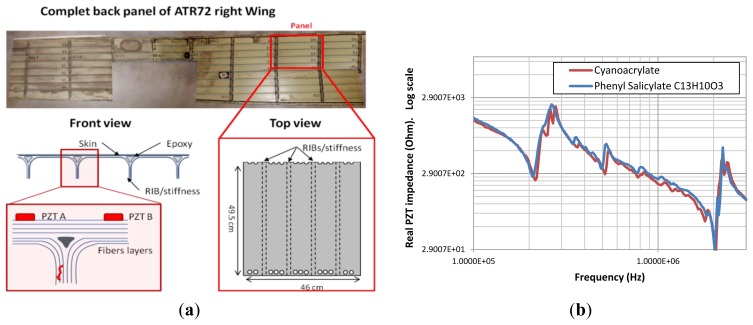
(**a**) Specimen of real aircraft structure part extracted from a right wing panel of an ATR72. (**b**) Influence of the pasting method on the PZT impedance response. The tests were done using cyanoacrylate glue and phenyl salicylate, respectively.

To demonstrate the feasibility of delamination detection in composite aircraft structures using our developed method, we artificially introduced three defects located at different zones using a calibrated impact machine. The impacts were applied from the upper face of the structure. The sensors presented in Figure 1(c) were pasted after the impact tests onto the upper side using a phenyl salicylate (C_13_H_10_O_3_) polymer that offers the unique advantage of polymerizing at 40 °C and depolymerizing at 60 °C, which is lower than the PZT material's Curie temperature. In other words, we can stick and remove our sensors without affecting their piezoelectric proprieties. Moreover, measurements (Figure 7(b)) show that the phenyl salicylate polymer offers the same coupling between the sensor and the structure as cyanoacrylate glue. As presented in Figure 7(a), an aircraft specimen of 49.5 cm × 46 cm was extracted from the right wing panel of an ATR72.

**Figure 8.**
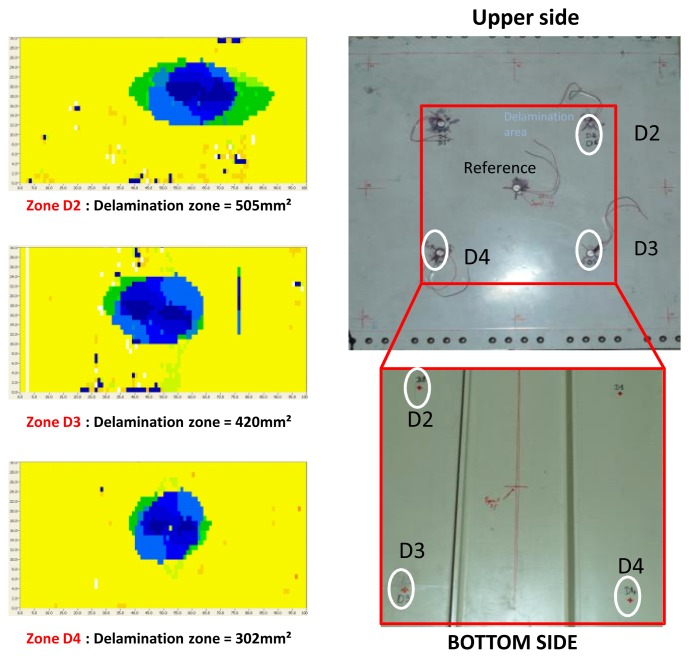
C-scan of the introduced delaminations, D2 area = 505 mm^2^, D3 = 420 mm^2^, D4 = 302 mm^2^ and photos of the upper and bottom face of the structure with mounted sensors.

A C-scan using a commercial NDT tool was performed to check the integrity of the structure and to quantify the artificially introduced damages' sizes. The damage D4 is situated deep inside the structure and is totally invisible from the upper or bottom side. The damages D3 and D2 are visible from the bottom side, while still totally invisible from the upper side. For high energy impacts, this phenomenon is typical behavior of composite structures (*cf.* Figure 8).

**Figure 9.**
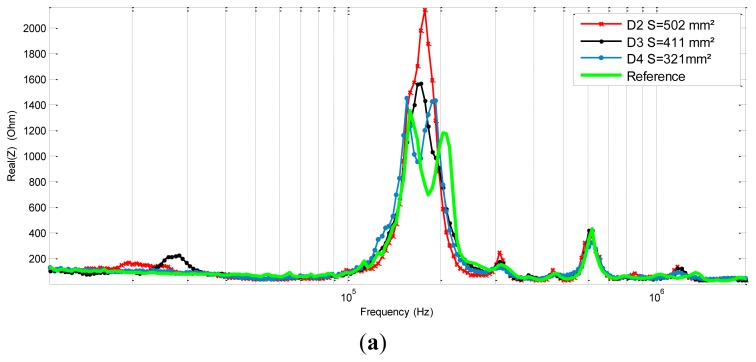

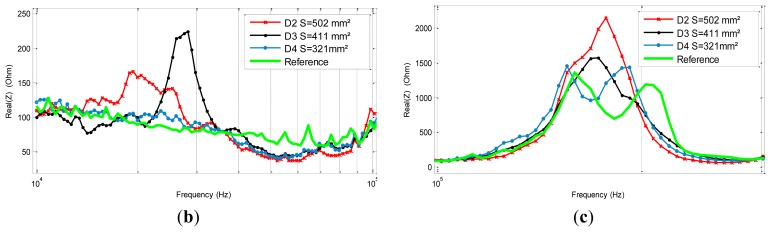
(**a**) Real part of the impedance measurement for each sensor. (**b**) Zoom onto lower frequencies [10 kHz–100 kHz]. (**c**) Zoom onto the sensors resonance frequency [100 kHz–300 kHz].

As shown in the literature [17,26,27] the electromechanical impedance technique is, contrary to modal analysis, efficient at higher frequencies (10 kHz–600 kHz). In this range, the wavelength of the excitation is very small, which allows for an accurate detection. The real part impedance spectra of the PZT sensors show different responses at many intervals. These differences seem to be more significant at lower frequencies but also around the first resonance and anti-resonance first mode of the piezoelectric sensor (*cf.* Figure 9). In this interval, we can clearly notice a peak shift and amplitude change. It is also interesting to see that for the reference signature and for the D4 response the signals have two main spikes between [100 kHz, 300 kHz] while the others have only one.

### 3.3. Limits of the Proposed Method

Using the test bench presented in Figure 4(b) we demonstrated the feasibility of PZT impedance reconstruction through the measurement of the consumed current and its voltage response. Although, the cost is low, the presented test bench setup it still heavy and unsuitable for a real integration. The method is also time-consuming because of the FFT that we used to perform the complex impedance calculations. A more integrated system should therefore be developed to avoid the use of laboratory instruments.

## 4. Toward a System on Chip (SoC) EMI integration

### 4.1. Implementation of the Method into the Embedded System

After we demonstrated the feasibility of E/M impedance calculation using a linear chirp excitation signal, we propose to miniaturize the test bench presented in Figure 3(b) using an embedded system. The the Agilent 33220A laboratory instrument was replaced by a programmable single scan waveform generator chip, the AD5932 manufactured by Analog Devices capable of providing a pure sine wave output signal with a linear frequency sweep increment up to 25 MHz [28].

**Figure 10.**
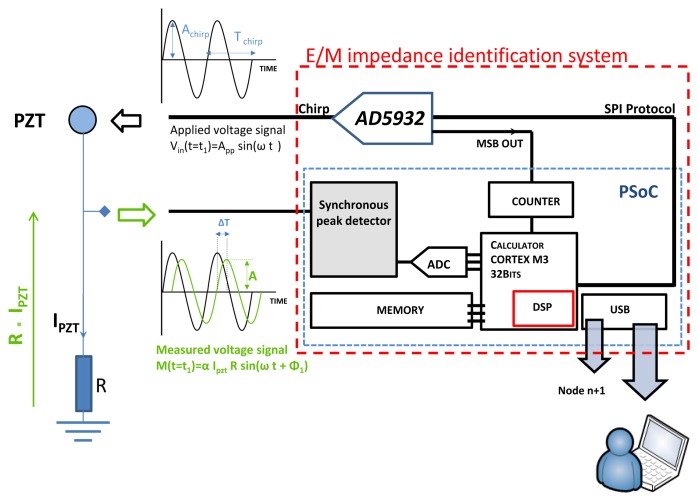
Synoptic of the developed E/M impedance calculation technique. Note that R is equal to 10 Ω; ΔT is the time delay between the excitation signal and the sensor response.

The PCI NI acquisition card plus a part of the algorithm that runs to the computer of Figure 4(b) is replaced by a reconfigurable system-on-chip (PSoC 5) provided by Cypress © to perform the signal conditioning, the acquisition and finally the impedance calculation (*cf.* Figure 10). The PSoC5 actually includes a reconfigurable analog part composed of comparators, operational amplifiers, mixers, transimpedance amplifiers two SAR and one sigma delta analog two digital converter plus other analog parts. It also includes an ARM CORTEX 32 bits CPU and a complex programmable logic device unit that could be programmed in Verilog [29]. Many communication modules and protocols like the USB full speed are also provided. The programming of the chip includes C language, Verilog [29] as well as analog schematic drawing.

**Figure 11.**
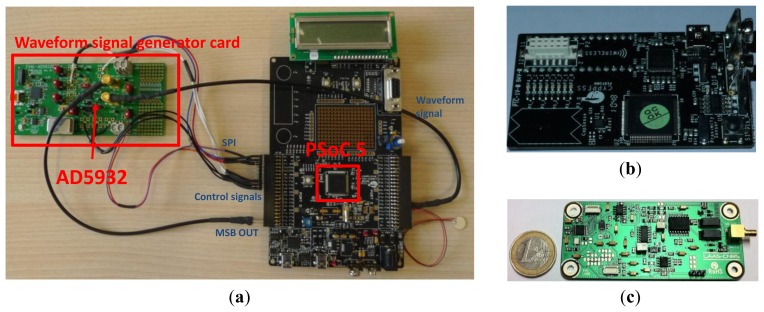
(**a**) Photo of the implemented hardware. (**b**) and (**c**) are respectively the PSoC5 card and the analog conditioning card that includes an AD5932, a charge amplifier, an analog filter and switching circuits. The two cards present the miniaturization of the complete hardware presented in (a).

Some integration methodologies were already described in the literature [26]. They generally use a very powerful solution based on Digital Signal Processing (DSP) to perform the impedance calculation. However, this solution is not optimal for embedded applications because of the high power consumption of DSPs and the scaling of the system which should remain low. The integration of the EM impedance was initially performed using the hardware presented in Figure 11(a). The complete system has a size of 15 cm−10 cm. After a miniaturization step the system scaling was further reduced to 7 cm × 4 cm.

#### 4.1.1. Waveform Stimulus Generation

**Figure 12.**
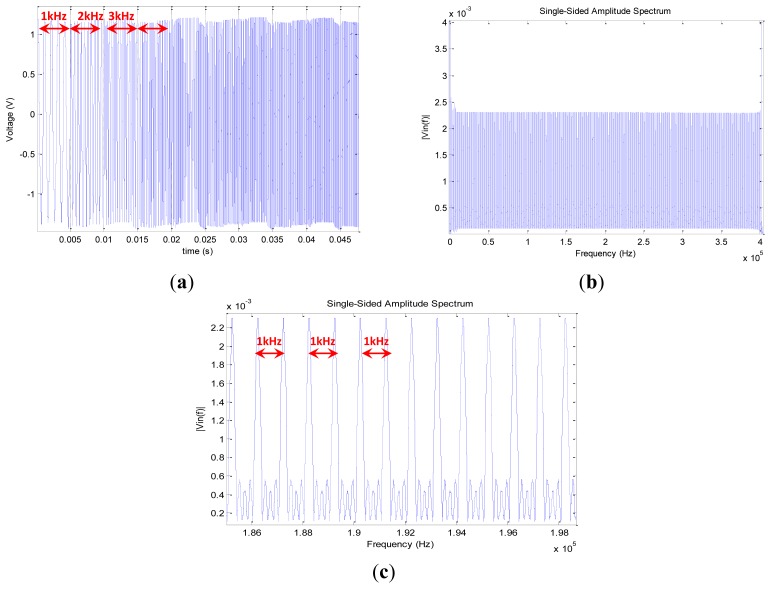
(**a**) Zoom onto the excitation signal. Notice that we can distinguish after each 5 ms the incrimination of the frequency. (**b**) Spectral response of the excitation frequency. (**c**) Zoom onto the spectral response of the excitation signal between 186 kHz and 198 kHz. Notice that the spikes are equidistant and have the same amplitude.

As presented in Figures 10 and 11, the applied stimulus voltage signal is generated by the AD5932, which was programmed using an SPI protocol to output a sine wave signal going from 1 kHz to 400 kHz with a step size of 1 kHz. The frequency increase is automatic and occurs after each 5.1 ms. Finally, the excitation signal lasts 2.05 s. In opposed to the chirp generated using the Agilent 33220 presented in Figure 5, the excitation signal generated by the AD5932 presents a non-continuous frequency variation (*cf.* Figure 12(a)). Because of the implemented incrementation of 1 kHz, the spectral response has a spiked shape (*cf.* Figure 12(c)).

#### 4.1.2. Impedance Calculation Using the System on Chip

Unlike for the previous method presented in the Figure 4(b) that is based on laboratory instruments and on a computer with a powerful CPU, the calculation of the impedance in this case should not be based on a fast Fourier transform to meet the specifications of embedded systems. Because of the limited resources of the CORTEX M3 CPU included in the PSoC, the impedance is calculated using the polar representation of the current and the voltage for each incremented frequency:

(7) $$I_{\text{PZT}}(f=f_{1})=|I_{\text{PZT}}(f_{1})|e^{i(2\pi f_{1}.t+\varphi )}$$

(8) $$V_{\text{PZT}}(f=f_{1})=|V_{\text{PZT}}(f_{1})|e^{i(2\pi f_{1}.t)}$$

(9) $$Z_{\text{PZT}}(f=f_{1})=\frac{\left|V_{\text{PZT}}(f_{1})\right|}{\left|I_{\text{PZT}}(f_{1})\right|}$$

**Figure 13.**
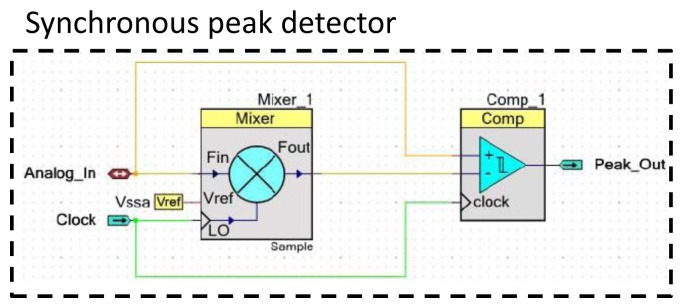
Schematic of the synchronous peak detector.

**Figure 14.**
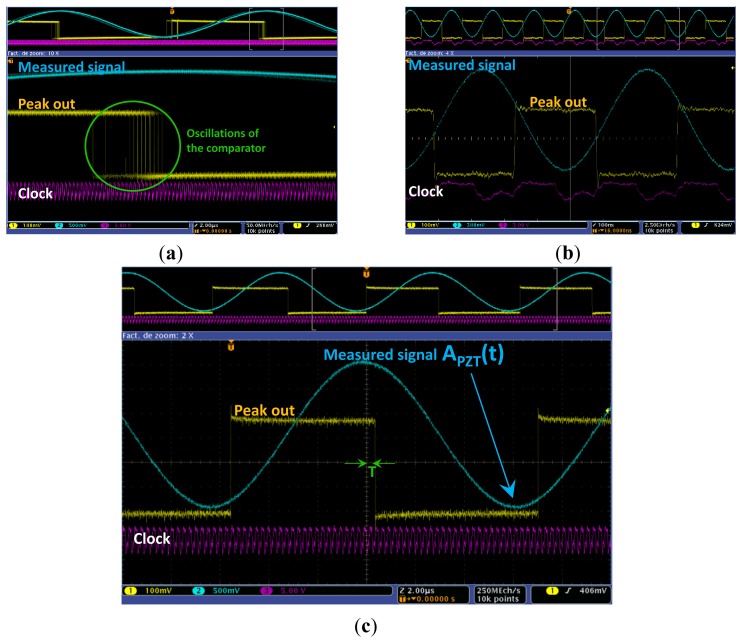
Experimental results acquired using an oscilloscope for the implemented peak detector. Notice that the clock is fixed to 4MHz for the three experimentations. (**a**) The frequency of the injected signal is too slow (8 kHz) for the used clock. Notice the oscillations of the comparator. (**b**) The injected signal is too fast. Notice that its frequency (2 MHz) is half the used clock frequency. The detection is totally erroneous. (**c**) Correct detection due to a ratio of 50 between the injected signal and the master clock.

Using the same circuit presented in Figure 4(a), we measure |*I_PZT_*(*f*)| using a synchronous peak detector presented in Figure 13 to identify for each frequency increment the maximum amplitude *A_PZT_* (*f*) of sensor response. When the output of the peak detector reaches a low state (*cf.* Figure 14), it triggers an ADC that acquires the value of the signal peak amplitude. Finally, the current response modulus is calculated for each frequency using this equation:

(10) $$|I_{\text{PZT}}(f)|=\frac{A_{\text{PZT}}(f)}{R_{c}}$$

where *A_PZT_* (*f*) is the maximum amplitude of the acquired signal and *R_c_* = 10 Ω the value of the measurement resistance presented in Figure 4(a). According to circuit mesh method, the sensor voltage response |*V_PZT_*(*f*)| is calculated by a simple subtraction for each MSB OUT front (triggered when the excitation voltage is high) between the excitation signal that is delivered by the AD5932 and the measured *A_PZT_*(*f*).

The synchronous peak detector comprises a down-mixer acting as a sample and hold block followed by a comparator with approximately 10 mV of hysteresis. The sample and hold induces a time delay on the input signal. This signal is afterwards compared using the hysteresis comparator to the original signal. As presented in Figure 13, the time delay created by the down-mixer is fixed using a selected sample clock. Its value is critical because it fixes the resolution of the detection. A clock that is too fast will create erroneous triggers due to oscillations at the comparator output (*cf.* Figure 14(a)). However, the peaks are missed when the sampling clock is too slow (*cf.* Figure 14(b)).

The correct clock should be fixed according to the frequency of the measured signal. As presented in Figure 14(c), the correct ratio was fixed at 50f, where f is the frequency of the input signal. The 10 mV hysteresis of the comparator is needed to ensure that noisy signals will not cause an oscillation in the comparator output.

**Figure 15.**
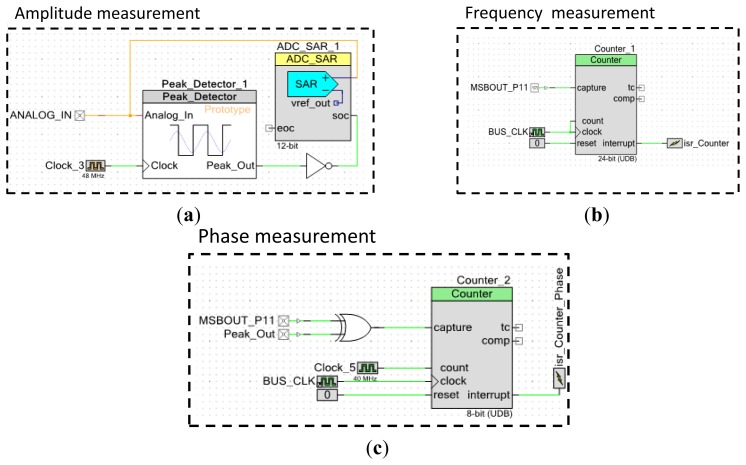
(**a**) Amplitude measurement stage: the peak detector output triggers the ADC acquisition. (**b**) Frequency measurement feedback stage measures the period of the MSB OUT signal that is generated by the AD5932. It allows a period precision control of 0.66 ns. (**c**) Phase calculation using XOR gate. The minimum measurable ΔT between the excitation signal and the measured one is equal to 0.25 ns.

Even if it is not used, the phase shift between the voltage and the current is calculated easily. For each frequency increment, the AD5932 signal generator emits a square signal through its MSB OUT pin that is synchronized with the generated signal and which has the same period of the pure sine generated wave. Therefore, to determine the phase of the current I_PZT_ we only have to apply an XOR operation between the square signal generated by the peak detector and the MSB OUT signal (*cf.* Figure 15(c)). The XOR allows the determination of the ΔT between the excitation signal and the sensor response. It is presented in Figure 15(c). The phase is calculated using this simple relation:

(11) $$\Delta T=\frac{\varphi }{2\pi }$$

To have a feedback of the AD5932 applied frequency, we used a 24 bits resolution counter triggered by the bus clock of the chip (48 MHz) which gives a resolution of 2Hz (*cf.* Figure 15(b)). The acquired data is afterward stored into a dynamic array situated in the flash memory of the PSoC5 and transferred to a computer using an USB connection where Matlab is installed.

**Figure 16.**
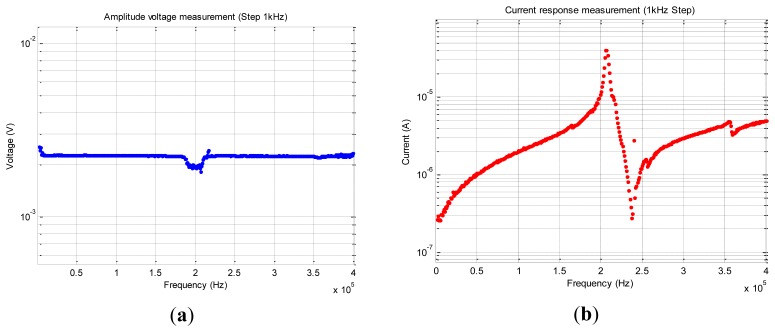
(**a**) V_PZT_(f): Measurement of PZT voltage amplitude after each frequency incrementation. The measurement was performed between 0 and 400 kHz with a step of 1kHz (**b**) I_PZT_(f): Measurement of PZT current response amplitude for each frequency incrementation between 0 and 400 kHz.

**Figure 17.**
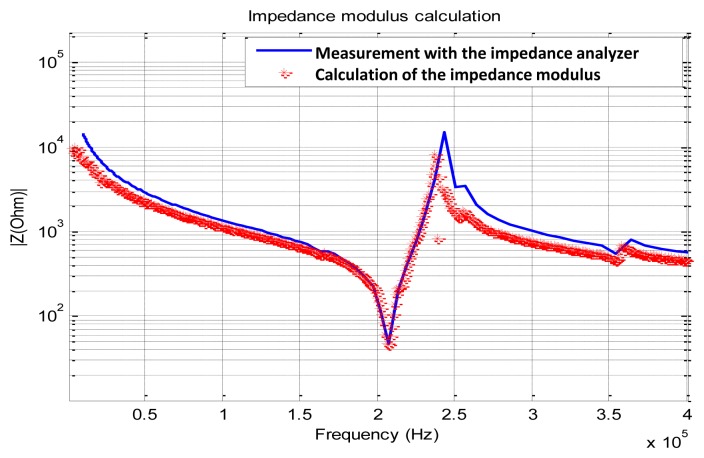
Comparison between the calculated impedance using signals of Figure 9(a,b) setup and the one measured using an Agilent 4294A impedance analyzer.

For an excitation signal going from 1 kHz to 400 kHz with a step of 1 kHz (*cf.* Figure 12), we record 399,999 acquisition points that are represented in Figure 16. Using Equation (9) we calculate the modulus of the impedance and we plot it in Figure 17, where the result is compared to the one measured using the Agilent 4294A impedance analyzer. Notice that our embedded proposed method is quite accurate; we register some erroneous points due some unexpected oscillation output of the comparator stage into the synchronous peak detector.

The complete implemented EMI calculation method with the stimulus waveform control code gives a total occupation of 36.9 kbytes which corresponds to 14.1% from the total available PSoC Flash memory. The total used SRAM is 4.7kbytes, which is only 7.3% of the total available cells.

### 4.2. Flaw Detection Using the Embedded EMI on Aircraft Composite Structure Stiffeners

**Figure 18.**
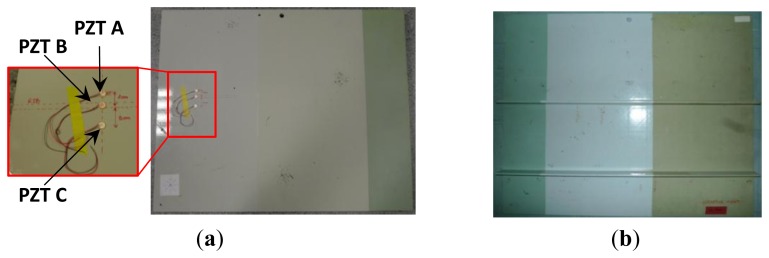
Wing front spar specimen (**a**) Front side view of the structure with PZT A at 2 cm from the rib, PZT B pasted onto the rib and PZT C at 3 cm from the rib (**b**) back side of the structure.

In order to demonstrate the detectability of mechanical flaws in composite structures stiffeners using the E/M impedance method we extracted from a wing front spar a part of 80 cm × 64.5 cm × 0.8 cm size that includes two ribs of 4 cm (*cf.* Figure 18). The idea is to detect damages insides these critical zones using sensors pasted onto the external surface of the structure. In other words, the sensors have no direct contact with the stiffeners.

**Figure 19.**
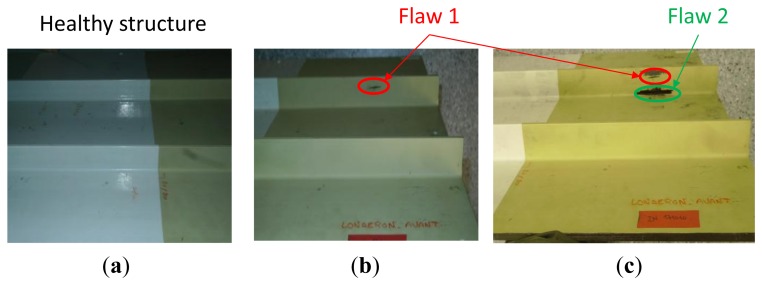
(**a**) Healthy wing front spar. (**b**) The same structure with one introduced crack into the second stiffener. (**c**) The same stiffener with a second introduced crack.

**Figure 20.**
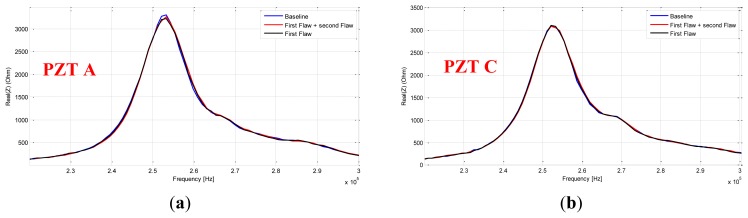

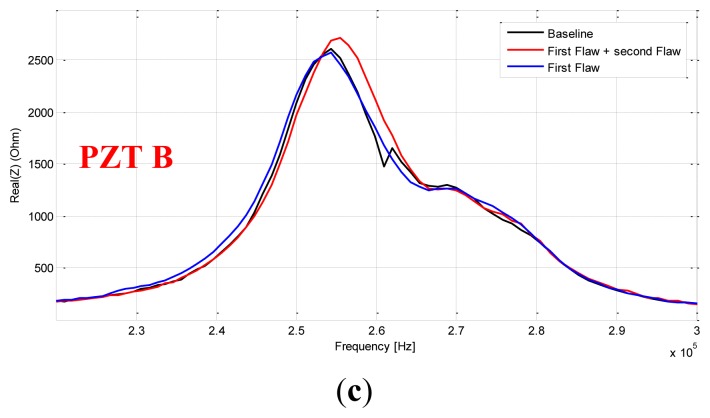
Real part impedance spectrum [220 kHz; 300 kHz] for the healthy signature, the response with one flaw and the response with two damages (**a**) superposed responses for PZT A. (**b**) Superposed responses for PZT C. (**c**) Superposed responses for PZT B.

As before, our diagnosis principle is based on a comparison between a healthy response considered as the baseline and other responses captured after damaging events. As seen in Figure 18(a) we used three PZT patches pasted onto the front side of the structure at 2 cm above the stiffener, at its position and finally at 3 cm under its position. Two damages were artificially introduced using a miniature circular saw to simulate cracks. We captured the sensors' response after and before the introduction of each defect. As depicted in Figure 19, the first crack is at 3 cm from the skin and has a size of 2 cm and the second one has a size of 3.5 cm and is at 0.5 cm distance from the structure skin. All the damages were introduced before the attachment of the sensors. The idea behind this to make a system that is easily mountable and removable. Because of certifications issues the idea is to manufacture a system cable to probe some critical parts in a plane on the ground after a fixed hours of flights. Although, we focus our studies on a narrow band of frequency [230 kHz, 300 kHz] to reduce the amount of the processed data and thus makes the inspection time more efficient. The measurements were performed 10 times for each sensor. If we compare the responses of each PZT at the interval range going from 220 kHz to 300 kHz, we notice that the impedance spectrum responses of the sensors A and C stay almost unchanged after and before the introduction of the two damages (Figure 20). However, the responses of the PZT B show frequency shift between 250 KHz and 270 KHz. We also notice that spectral impedance value of the PZT B is linked with the health of the structure as in the case of delaminations.

## 5. Conclusions/Outlook

The work reported in this paper has shown how with a basic PZT sensor we can detect damages in aircraft composite materials using an inexpensive method through the E/M impedance spectrum analysis. We demonstrate the feasibly of structural health monitoring using non-permanently attached PZT patches and we presented the unique advantage and flexibility that the programmable system-on-chip technology provides to the E/M impedance method integrability. However, to cover larger areas and monitor a complete aircraft wing additional work will need to be carried out in order to reduce the system size and make this promising technique suitable for larger sensor networks. The challenges will be focused in the efficiency of the method for thicker zones of the planes ∼10 cm. The combination of the E/M impedance technique with another detection method based on modal frequencies analysis will also be considered.
